# Easy phylotyping of *Escherichia coli via* the EzClermont web app and command-line tool

**DOI:** 10.1099/acmi.0.000143

**Published:** 2020-06-19

**Authors:** Nicholas R. Waters, Florence Abram, Fiona Brennan, Ashleigh Holmes, Leighton Pritchard

**Affiliations:** ^1^​ Department of Microbiology, School of Natural Sciences, National University of Ireland, Galway, Ireland; ^2^​ Information and Computational Sciences, James Hutton Institute, Invergowrie, Dundee DD2 5DA, Scotland; ^3^​ Soil and Environmental Microbiology, Environmental Research Centre, Teagasc, Johnstown Castle, Wexford, Ireland; ^4^​ Cell and Molecular Sciences, James Hutton Institute, Invergowrie, Dundee DD2 5DA, Scotland; ^5^​ Strathclyde Institute of Pharmacy and Biomedical Sciences, University of Strathclyde, Glasgow, G4 0RE, Scotland

**Keywords:** phylogroups web app, classification, bioinformatics, genomics, *Escherichia coli*

## Abstract

The Clermont PCR method for phylotyping *Escherichia coli* remains a useful classification scheme even though genome sequencing is now routine, and higher-resolution sequence typing schemes are now available. Relating present-day whole-genome *E. coli* classifications to legacy phylotyping is essential for harmonizing the historical literature and understanding of this important organism. Therefore, we present EzClermont – a novel *in silico* Clermont PCR phylotyping tool to enable ready application of this phylotyping scheme to whole-genome assemblies. We evaluate this tool against phylogenomic classifications, and an alternative software implementation of Clermont typing. EzClermont is available as a web app at www.ezclermont.org, and as a command-line tool at https://nickp60.github.io/EzClermont/.

## Introduction

*Escherichia coli* is one of the most widely studied and best-understood organisms in biology. Even before widespread whole-genome sequencing, it was known that the *E. coli* species group is very diverse [1, 2], and several methods were developed to differentiate the various *E. coli* lineages. In 1987, Selandar and colleagues first used electrophoretic analysis of a 35 enzyme digest to classify the *Escherichia coli* Reference Collection (ECOR) into six groups (A–F) [2]. Subsequently, Clermont and colleagues published a triplex PCR method for phylotyping in 2000, able to differentiate four of these groups – A, B1, B2 and D [3]. In 2013, Clermont and colleagues updated this scheme, adding a fourth set of primers to detect groups E and F; additional primers were also proposed to differentiate the cryptic clades [4]. This method was again recently extended to include primers that differentiate the newly identified G phylogroup [5]. The Claremont quadruplex primers have been widely adopted for laboratory-based classification as the method is reliable, easy to interpret and correctly classifies about 95 % of *E. coli* strains.

Other typing schemes developed to classify *E. coli* strains include: Achtman seven-gene multilocus sequence typing (MLST) [6, 7]; Michigan EcMLST [8]; whole-genome MLST (www.applied-maths.com/applications/wgmlst); core-genome MLST [9]; two-locus MLST [10]; and ribosomal MLST [11]. All of these sequencing-based methods classify *E. coli* with greater accuracy and to higher resolution than Clermont phylotyping. Any practical choice of approach involves trade-offs of cost and complexity against the precision offered by the methodology. The Clermont phylotyping scheme [4] remains a popular tool for *E. coli* classification, as it can be performed rapidly and inexpensively in a laboratory. In addition, this classification scheme remains useful to make comparisons of newly sequenced isolates against historical literature, which contains many references to strains classified only by the Clermont scheme.

EzClermont was developed to bridge the gap between the traditional quadruplex primer approach to phylotyping and whole-genome sequence data. It provides a simple *in silico* analogue of the Clermont phylotyping approach, applied to genome assemblies. We implemented EzClermont as both a web application for public use, and as a command-line program for local installation. A similar tool called ClermonTyping was recently published, with similar goals and functionality [12]. Here, we describe our implementation of the Clermont classification scheme in EzClermont, assess its ability to correctly assign Clermont type, relative to *E. coli* whole-phylogeny, and compare its performance to the ClermonTyping program.

## Methods

### *In silico* PCR

To emulate PCR *in silico*, EzClermont uses regular expressions (regexes) that represent the Clermont primer sequences to locate their potential binding sites on a sequenced genome. The sequence regions lying between these sites are taken to be the predicted amplicons, and can be evaluated for sequence composition or presence/absence to determine Clermont phylotype.

In practice, PCR primer sequences do not require exact genomic matches to function, so primer-binding sequence variability must be captured in the corresponding regexes. To represent this variability, we selected 1395 *E. coli* genomes from EnteroBase [13] (accessed April 2019). After filtering genomes based on metadata quality and source, one representative of each Achtman seven-gene multilocus sequence type was selected. The list of 1395 isolates can be found in the EzClermont repository (https://github.com/nickp60/EzClermont/blob/master/docs/analysis/training/enterobase_training_subset.tab) (a detailed description and script of this filtering procedure can be found in the online repository under https://github.com/nickp60/EzClermont/blob/master/docs/analysis/3602-processing-Enterobase-metadata.Rmd).

The theoretical amplicons of each of the quadruplex, E-specific, C-specific, G-specific and E/C control primer sets were identified and aligned. Canonical sequences of the target alleles were identified from the National Center for Biotechnology Information database (Table 1). Primer sites were identified and the sequences extracted from the corresponding genomes, including an additional five nucleotides at the 5′ and 3′ ends. Homologous sequences were identified in each of the 1395 assemblies using reciprocal blast and the simpleOrtho tool (https://github.com/nickp60/simpleOrtho). Matching sequence regions were extracted and aligned using Mafft 7.455 [14], enabling reverse-complement hits with the -adjustdirection argument (other arguments were left as defaults). The resulting multiple-sequence alignment was used to identify variations at the canonical primer binding sites; these variations were incorporated into the primer sequence, represented as regular expressions (Table 1). Sequence variations in the last five bases of the primers were not incorporated into the regular expressions, as these 3′ variations can be used to differentiate alleles [15].

**Table 1.** Primers from the studies by Clermont and colleagues in 2013 and 2019 [4, 5] Target amplicons were identified from canonical genes (or intergenic regions). Ambiguities determined by the training procedure were incorporated as degenerate primer sequences using standard IUPAC (International Union of Pure and Applied Chemistry) codes, which are translated into regular expressions by EzClermont. Variations occurring in the final five bases of the 3′ ends of the primers were not incorporated.

Primer	Target gene	Canonical	Degenerate primer (5′→3′)
AceK_f	*aceK*_*arpA*	NC_000913.3: 4218596–4222487	AAYRCYATTCGCCAGCTTGC
ArpA1_r			TCTCCMCATACYGYACGCTA
chuA_1b	*chuA*	NC_011750.1: c4160640–4158658	ATGGTACYGGRCGAACCAAC
chuA_2			TRCCRCCAGTRCCAAAGACA
yjaA_1b	*yjaA*	NC_000913.3: 4213234–4213617	YAAACKTGAAGTGTCAGGAG
yjaA_2b			ARTRCGTTCCTCAACCTGTG
TspE4C2_1b	*tspE4.C3*	AF222188.1	CACKATTYGTAAGRYCATCC
TspE4C2_2b			AGTTTATCGCTGCGGGTCGC
ArpAgpE_f	*arpAgpE*	NC_000913.3: 4220301–4222487	RATKCMATYTTGTCRAAATATGCC
ArpAgpE_r			GAAARKRAAAADAMYYYYCAAGAG
trpBA_f	*trpBA*	NC_000913.3: 1316416–1318415	CGGSGATAAAGAYATYTTCAC
trpBA_r			GCAACGYGSCBWKRCGGAAG
ybgD_F	*ybgD*	NZ_UIKK01000035.1	GTTGACTAARCGYAGGTCGA
ybgD_R			KATGYDGCYGATKAAGGATC
trpAgpC_1	*trpAgpC*	NC_000913.3: c1317222–1316416	AGTTYTAYGCCSVRWGCGAG
trpAgpC_2			TCWGYDCYVGTYACGCCC

### Validation dataset and phylogeny estimation

**Fig. 1.**
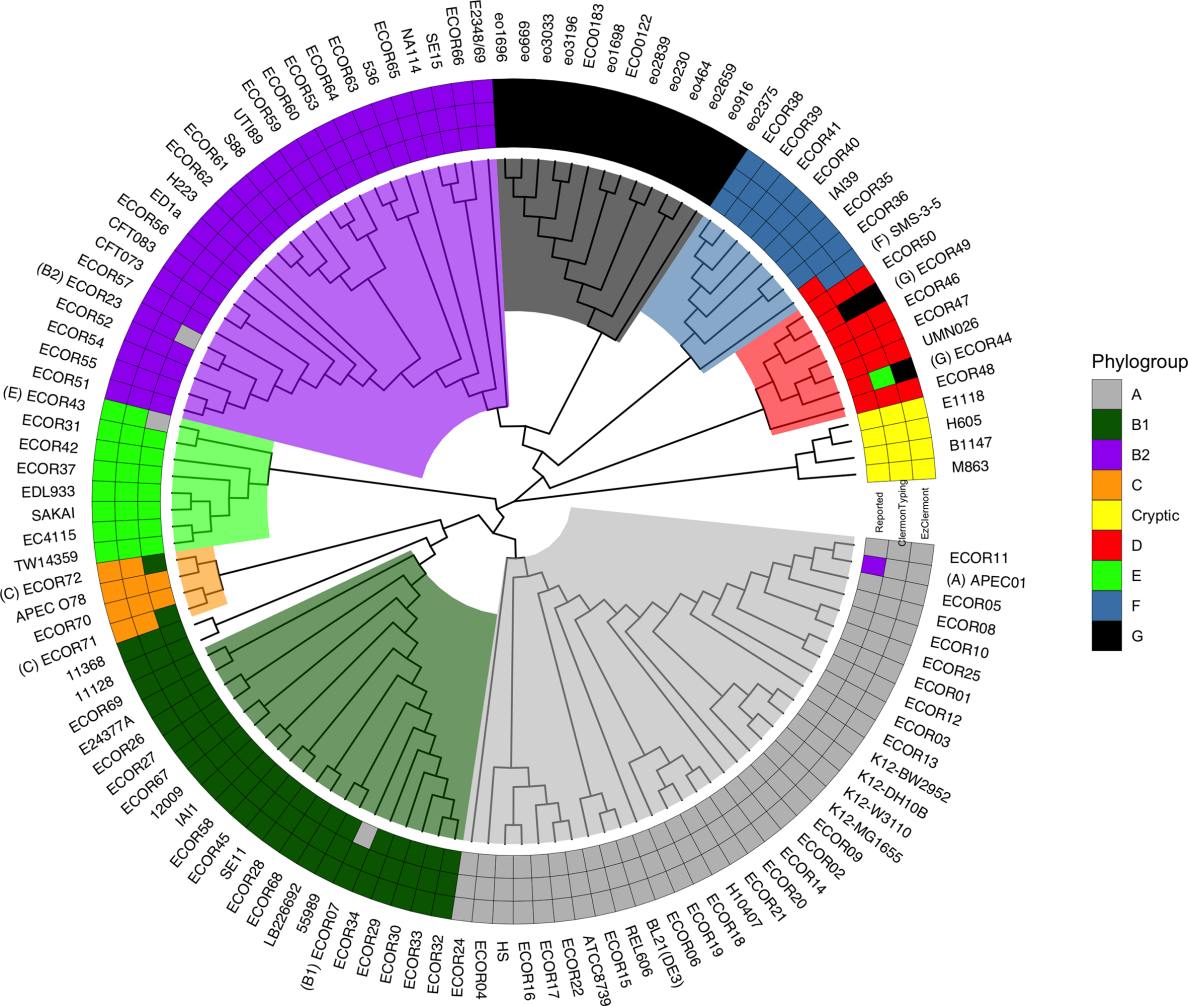
Cladogram of whole-genome phylogeny for members of the ECOR collection and phylogroup G isolates from the work by Clermont and colleagues [5]. Clades are background-coloured by dominant phylogroup. The heatmap surrounding the tree shows phylogroups determined from: literature (inner ring), ClermonTyping (middle ring) and EzClermont (outer ring). The literature phylogroup was not supported by *in silico* analysis for seven strains. Both EzClermont and ClermonTyping agree with the phylogenetic lineage in all but two cases: ECOR44 and ECOR49.

The accuracy of EzClermont classification was assessed against a set of 125 *E. coli* isolates having both experimentally determined Clermont phylotypes and available whole-genome sequencing data. The accession numbers for these 125 strains can be found in the GitHub EzClermont repository (https://github.com/nickp60/EzClermont/blob/master/docs/analysis/validate/validation_metadata.csv). We used Parsnp [16] to obtain a core-genome alignment for the 125 strains, similarly to the approach taken by Clermont *et al*. [5]. PhyML [17] was used to estimate the phylogeny of these strains using this nucleotide alignment as input and the HKY85 substitution model, obtaining approximate Bayes bootstrapped branch support. The resulting tree was visualized with ggtree [18], and branches were rotated so that the cryptic *Escherichia* assemblies initiate the tree (Figs 1 and S1, available with the online version of this article).

### *In silico* Clermont classification

Both EzClermont (version 0.6.2) and ClermonTyping (version 1.4.1) were run with default parameters on the 125 strains in the validation dataset.

## Theory and Implementation

EzClermont is an open-source Python package distributed under the MIT License, available via PyPI (https://pypi.org/project/ezclermont/), Conda and GitHub (https://github.com/nickp60/ezclermont). Biopython is utilized for parsing sequences [19]. The package comprises a command-line tool for batch execution and a Flask-based web app. The web app is hosted as a live service at http://ezclermont.org, and a Docker container is available at https://hub.docker.com/r/nickp60/ezclermont for local deployment.

### Performance

Performance of both tools was assessed on a MacBook Pro laptop with a 2.7 GHz Intel Core i7 processor with no other applications running apart from a terminal application. Five runs of the 125-strain validation set were analysed by each tool, and the elapsed wall time was recorded for each. Results are shown in Fig. S2.

## Results

Clermont types reported in the literature are not guaranteed always to correspond to phylogenetic lineage for *E. coli *; *in silico* predictions of phylotype may agree with reported type, lineage, both or neither. Therefore, we first established the correspondence between lineage and Clermont type for each isolate in the 125-member validation set and visualized this in Fig. 1. We found that for seven isolates, the lineage was not consistent with the recorded Clermont type (Table 2). In these cases, we considered that the phylogenetic lineage was more reliable and took precedence over literature-reported Clermont type for validating the *in silico* methods.

**Table 2.** Isolates with inconsistent phylogroup predictions EzClermont and ClermonTyping were run on a set of strains with reported phylotypes. A core SNP tree was reconstructed, allowing comparison between predicted and reported phylotypes, and the estimated phylogeny.

Strain	Accession no.	Reported	Phylogeny	ClermonTyping	EzClermont	Note
APEC01	GCA_003028815.1	B2	A	A	A	
ECOR07	GCA_003334305.1	A	B1	B1	B1	
ECOR23	GCA_003334095.1	A	B2	B2	B2	
ECOR43	GCA_003333775.1	A	E	E	E	
ECOR44	GCA_003333765.1	D	D	E	G	ArpA1_r G17A
ECOR49	GCA_003333685.1	D	D*	G*	G*	
ECOR71	GCA_003333385.1	B1	C	C	C	
ECOR72	GCA_003334425.1	B1	B1	C	C	
SMS-3–5	GCA_000019645.1	D	F	F	F	

*Both tools mistype ECOR49 types as phylogroup G due to a potentially contaminated assembly; ECOR49 from assembly GCA002190975.1 is correctly typed by both tools as phylogroup D.

Fig. 1 also summarizes the results of applying both EzClermont and ClermonTyping to the validation dataset. For 123 of 125 isolates, the *in silico* method predictions were consistent with the dominant Clermont type of the phylogenetic lineage. The two mismatched isolates ECOR44 and ECOR49 are, by lineage and literature report, phylogroup D, but were mistyped by both EzClermont and ClermonTyping as phylogroups G or E. We examined the source assembly for the ECOR49 isolate and found by reciprocal blast search that the canonical *arpA* fragment that should be present in phylogroup D could not be identified. This would be sufficient to cause misclassification, and suggested that the assembly used for validation might not be complete. We confirmed this by also analysing the alternative ECOR49 assembly GCA_002190975.1; this assembly contains the *arpA* fragment and both tools assigned this genome correctly to phylogroup D.

The ECOR44 isolate was mistyped by ClermonTyping as phylogroup E, and by EzClermont as phylogroup G. This was suggestive of a false-negative result *in silico* for the *arpA* primer set. Closer inspection of the region indicated that the *arpA* fragment was not correctly identified due to a G to A substitution at base 17 of the reverse primer binding site. This mutation occurs in the final five bases of the reverse primer, and so was not incorporated during the training process for the primer regular expressions; the same mutation was seen in a further 8 of the 1395 training isolates.

We ran our analyses on the 125 member validation set five times with both EzClermont and ClermonTyping (Fig. S2). The mean execution time was 1.74 s for EzClermont and 1.48 s with ClermonTyping.

## Discussion

EzClermont was built to bridge the gap between established laboratory and whole-genome sequencing methods of classifying *E. coli *. Both EzClermont and ClermonTyping correctly classified 123 of the 125 isolates in our validation set, indicating that they each perform with an approximately 98 % true-positive rate (TPR). Furthermore, a much broader application of EzClermont by Zhou *et al*. [13] to representative *E. coli* strains in EnteroBase was found to be strongly in agreement with both higher-resolution sequence typing and with ClermonTyping. EzClermont identifies only that isolates are classified as ‘cryptic’, where ClermonTyping distinguishes between cryptic lineages.

Both tools mistyped the same pair of isolates from the validation set. Incomplete assemblies and misassembled genomes, in particular, are always likely to give erroneous results with genome sequence-based methods. Input

genome quality is, therefore, critical for accurate classification. The *arpA* fragment appears to be particularly problematic, and Beghain *et al*. [12] noted the difficulty in typing with this region, which has likely been horizontally transferred to some phylogroup D isolates.

However, the disagreement observed in this study between phylogenetic lineage and literature-reported phylotype for seven isolates reinforces that laboratory assays also share potential for error, and that these errors may be propagated in literature and metadata. Our comparison of sequencing efforts for the same isolates in two BioProjects implies that, at least in these two collections, the phylogenetic identities of 12 of the 72 strains were not certain (Fig. S3). Such issues may lead to groups referring to distinct strains by the same name. We found that application of the *in silico* tools was able to correct misassigned phylotype for seven isolates.

EzClermont is implemented as an application and as a Python package, and works with STDIN/STOUT for developers to integrate into Unix pipelines. It is also presented as a web application with an intuitive interface for simple queries. We hope that the incorporation of EzClermont into EnteroBase [13], and the utility of applying the local program to large batches of genomes, mean that it will be of continued use to the scientific community.

## Supplementary Data

Supplementary material 1
