# Supplementary material for: Easy phylotyping of Escherichia coli via the EzClermont web app and command-line tool
Source: Access Microbiol. 2020 Jun 19;2(9):acmi000143. doi: 10.1099/acmi.0.000143 (PMC7656184; doi:10.1099/acmi.0.000143)
Supplement: Supplementary material 1 [file acmi-2-143-s001.pdf]

Supplementary Information

0.0.1 Generating core-genome alignments

Core genome alignments were generated with Parsnp (version 1.1.2) from a curated directory of genome assemblies, selecting a random reference, run with 4 threads, as follows:

```
parsnp -c -d ./genome_assemblies_genome_fasta/tmp/ -r ! -p 4 -o ./alignment/ harvesttools -i
./alignment/parsnp.ggr -M ./alignment/parsnp.msa
```

Harvestools was used to convert the resulting core genome alignment into a .MSA file, which was converted into a PHYLIP alignment with biopython as follows:

```
# names were truncated to retain only the unique identifier cat alignment/parsnp.msa | sed -e 's/^>\(.\{4\}\)\(.\{9\}\).*/
> alignment/parsnp.clean.msa
# from open_utils commit 65129b2
frommsa.py ./docs/analysis/validate/alignment/parsnp.clean.msa >
./docs/analysis/validate/alignment/pa
```

Phyml (version 3.3.20190909) was run as follows, using the default parameters for nucleotide alignments.

```
# version 3.3.20190909 phyml -i
./alignment/parsnp.clean.phy
```

0.0.2 Phylogenetic analysis of the ECOR collection sequencing efforts

0.0.3 Timing

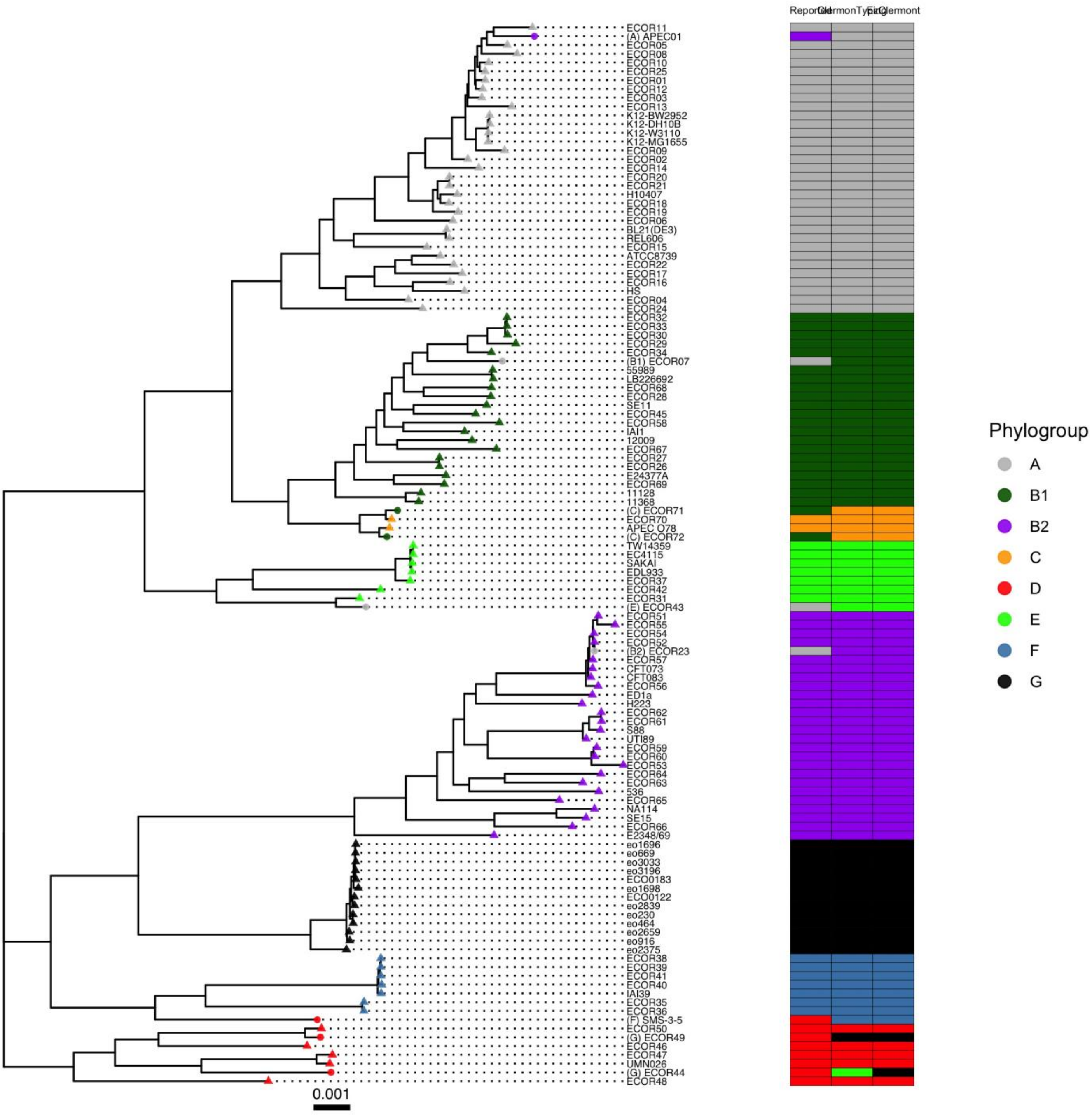

Figure S1: Maximum likelihood tree of core genome alignment showing the relationship of the isolates to their literature-reported and predicted phylotypes. The cryptic clade isolates were omitted for clarity, and the tree was rerooted at the point where they diverged.

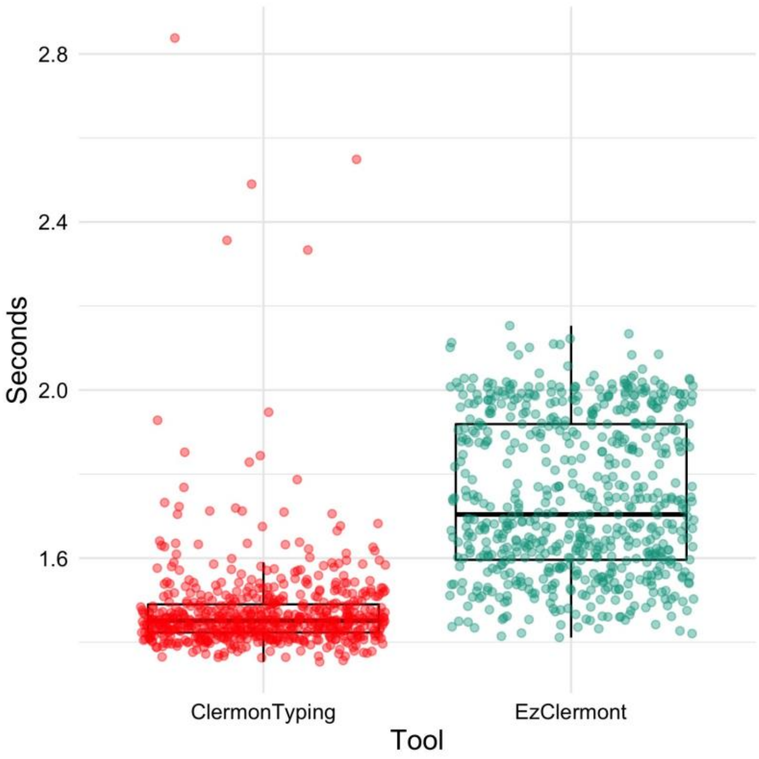

Figure S2: Timing was assessed by running each tool on the 125 validation strains 5 times, for a total of 625 runs. ClermonTyping has a slight time advantage compared to EzClermont.

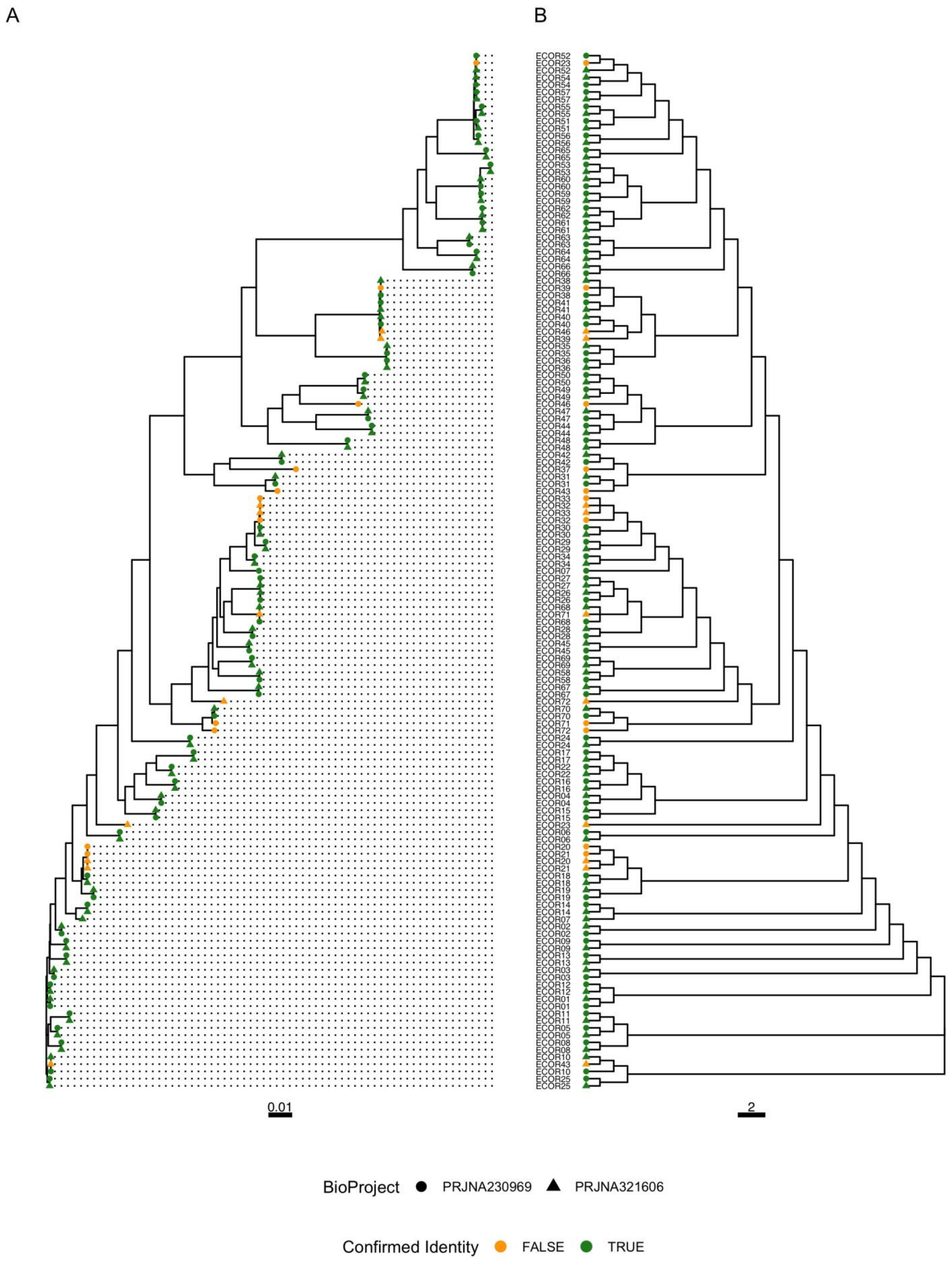

Figure S3: A core-genome alignment of the assemblies from both ECOR collection sequencing projects was created with Parsnp. The tip shapes indicate the sequencing project. (A) shows the core genome phylogeny, and (B) shows the same but with equal branch lengths to more easily visualize closely-related strains. Yellow tips indicate strains for which the two BioProject's assemblies contradict.
